# Factor Analysis of Quality Management Systems Implementation in Healthcare: An Online Survey

**DOI:** 10.3390/healthcare10101828

**Published:** 2022-09-21

**Authors:** Mustafa Rawshdeh, Heather Keathley, Shahed Obeidat, Raed Athamenh, Moayad Tanash, Dania Bani Hani

**Affiliations:** 1 Department of Industrial Engineering, Faculty of Engineering, The Hashemite University, P.O. Box 330127, Zarqa 13133, Jordan; 2 Department of Industrial Engineering and Management Systems, University of Central Florida, Orlando, FL 32816, USA; 3 Department of Mechanical and Industrial Engineering Applied Science Private University, P.O. Box 166, Amman 11931, Jordan; 4 Hijjawi Faculty for Engineering Technology, Yarmouk University, Irbid 21163, Jordan

**Keywords:** quality management systems (QMS), implementation success, healthcare, survey, exploratory factor analysis (EFA), linear regression (LR)

## Abstract

This paper investigates the views of healthcare researchers and professionals on the implementation of the Quality Management System (QMS) approach using a 5-point Likert scale survey. Researchers and healthcare professionals who observed or participated in QMS implementation were surveyed. Multiple channels, including occupational societies, social networking, i.e., LinkedIn, hospital’s directories, award recipients, academic researchers, and professional connections, made it possible to reach this particular sample. Participants were surveyed using a series of questions with a total of 56 questions. The survey was administrated through the web portal of Qualtrics and then analyzed both on Qualtrics and SPSS software packages. Descriptive Statistics, Exploratory Factor Analysis (EFA), and Linear Regression were employed to draw conclusions. The final sample group consisted of 71 participants representing a range of healthcare settings. EFA was conducted, producing a model of 10 emergent factors and an outcome for total improvement. Regression modeling revealed the Critical Success Factors (CSFs) and the interaction between emergent factors. The results indicated that QMS Implementation Culture, Structure, and Managerial Training are critical to the QMS implementation success. This research helps quality professionals enhance their ability to prioritize elements affecting the successful implementation of the QMS.

## 1. Introduction

The pursuit of adequate improvement of organizational processes, procedures, and policies has encouraged healthcare systems to seek out suitable quality management schemes [1]. Achieving a high level of service quality is essential for healthcare decision-makers to ensure the highest level of performance [2]. Healthcare organizations require a strategy to ensure high-quality work that is aligned with their vision and mission, thereby satisfying both internal and external customers [3,4,5,6]. This approach could enhance control over all processes and procedures [3]. As described by the Donabedian model, the quality of healthcare services is evaluated by the comprehensiveness of data from process, structure, and outcomes [7]. The foundation for achieving quality in healthcare services at all levels is by creating sustainable quality in line with the needs and demands of the customers [8,9].

In healthcare, policymakers’ choice to utilize the Quality Management System (QMS) requires the use of proper success measures [10]. Researchers have used the implementation factors to achieve those measures [6]. Quality requires high standards of compliance. The American Society of Quality (ASQ) defines QMS as permanent systems that plan and organize the quality in each process [11]. The primary goals of QMS are to align quality with the organization’s specific vision and mission, satisfy external and internal customers, and achieve higher performance and business improvement [4]. Specific requirements and standards defining quality values and objectives that support a system are built on some well-established standards, such as the International Standards Organization (ISO 9001) or quality models, such as the European Foundation for Quality Management (EFQM) model. Moreover, healthcare dedicated certification requirements could define quality values and objectives that support a system, such as Joint Commission International (JCI) [12].

In complex industries, such as healthcare, quality management is an interdisciplinary process. The inherent complexity of healthcare quality was acknowledged in various reviews of improvement initiatives [13,14]. Parasat et al., 2019 concluded five distinct dimensions of healthcare quality complexity [15]. The first dimension is heterogeneity, which is exemplified by the high level of individualized care due to patient-specific treatment pathways. The second dimension is the gap between the knowledgeable practitioner and the patient. The third dimension is that patients and healthcare providers are exposed to high risks and costs associated with the services provided, where failure is considered to have a high cost. The fourth dimension consists of the stringent regulations that govern healthcare organizations. Finally, the lengthy duration of service delivery involving multiple treatment modalities may influence patients’ perceptions of the quality of care. Therefore, healthcare leaders must have an in-depth understanding of quality concepts, the implementation of quality within systems, and the relationships within healthcare organizations [16].

Multiple studies have empirically shown that successful QMS implementation is linked to improved clinical outcomes, such as mortality, complications, and patient safety, and administrative outcomes, such as the average length of stay, profitability, and expenses incurred per discharge [17,18]. Aburayya, Alshurideh [19] found an empirical connection between TQM practices and a higher level of service quality, namely, higher degree of conformance to service specifications or requirements. The previous results suggest a potential effect of QMSs on multiple healthcare dimensions. Despite the promising benefits of adopting QMSs in healthcare, many studies have reported difficulties during implementation or unsatisfaction with the resulting system [1,20,21,22].

Critical Success Factors (CSFs) include strategies and approaches that represent the implementation structure or a way to conduct things [23]. The success factors present a set of areas that, when applied and reinforced, provide a competitive advantage for organizations to achieve their goals [24]. CSFs consist of strategies and approaches, signify implementation structure or a method of conducting things [24]. When applied and reinforced in an organization, the success factors comprise a set of areas. This provides organizations with a competitive advantage in achieving their objectives, but they must be aware of each factor [25]. Identifying the factors is a key element in ensuring the success of a system or a project. They are elements theorized to significantly affect the success of the implementation process [5,6]. For QMS implementation in healthcare, multiple studies are trying to report success factors for implementation along with reporting various factors. The types of factors were not unexpected, for example, studies have mentioned the customer focus approach as a success factor [25]. This factor conforms to the nature of QMSs, which have been designed to focus organizations on customer requirements. Other factors included leadership as the most important factor for implementation success with multiple sub-factors, such as management commitment and management training [6]. Factors, such as quality planning, education, continuous improvement, communication, and employee involvement, have also been heavily studied in regards to QMS success [26].

A Systematic Literature Review (SLR) by Rawshdeh et al. [27] revealed that investigation into the implementation of success factors in healthcare was mostly qualitative. Few studies used advanced quantitative techniques, such as correlation and factor analyses, to analyze implementation success factors. In addition, the literature has not empirically tested the relationship between implementation factors and success outcomes. The previous quantitative analysis revealed a variation in the studied factors, their terminology, and the studies’ context. It should be noted that all factors are directly related to the principles of different models of QMSs. Many of the identified factors have significant variations in the categories studied and terminology. This suggests that a comprehensive model is needed to evaluate the effects of the factors as well as identify the CSFs [5,26]. The results can provide the literature with a robust model for implementation success that can effectively enhance the implementation experience making the potential benefits of QMSs available to more organizations.

In this area of research, there was a lack of concrete empirical evidence for factors’ structure and the relationship between factors and outcomes. Consequently, there is a need for modern research based on a comprehensive understanding of QMSs and advanced empirical analyses. This research aims to develop a robust construct of factors and provide the necessary relationship analysis between factors, resulting in a comprehensive framework of factors and outcomes.

## 2. Materials and Methods

A survey instrument was constructed to refine multi-item constructs that can be used to quantify the effects of the factors on implementation outcomes and to investigate relationships among factors and outcomes.

### 2.1. Operational Research Model Development

An Operational Research Model was developed by integrating the factors and outcomes discovered by Rawshdeh [27]. The result provided a structured list of factors and outcomes synthesized from evidence available in the literature and expert insights [27]. Factors and outcomes in this step were categorized into major groups: Management, culture, and structure. The research synthesis generated an extended list of factors that have been studied in the literature in the last few decades, which are integrated with contemporary factors provided by experts. Table 1 and Table 2 show the groups of factors and outcomes along with their items and frequencies.

The approach to establishing content validity involves literature reviews alongside expert evaluation. This survey used constructs with content validity since they were derived from an extensive review of the literature, consisting of multiple reviewers to ensure the constructs’ validity, alongside expert insights to ensure they are valid [28]. The resulting constructs and their sub-constructs, which are the sub-concepts within each factor, were used to create the Likert-scale items of the survey. Full details about the construction development can be provided upon request.

The next section discusses the use of Likert-scale survey questionnaire to refine the factors and ensure a robust model structure. In addition, it provides an analysis of the emergent factors’ connections to the achievement of the outcomes as well as the discovery of the inter-relationships among them.

### 2.2. Survey Design and Exclusion Criteria

The survey was designed to be taken online using the Qualtrics platform developed by Qualtrics company copy right version July 2020 Provo, Utah, USA. The survey consists of two sections. The first section focused on background information and contained nine questions that collected information about the respondents’ backgrounds. The information included position, years of experience, the type of QMS implemented, the overall level of implementation success, and the size and type of healthcare organization. Moreover, it included the exclusion criteria represented by questioning the participation in QMS implementation in healthcare, and their role in the implementation. These questions aimed to filter participants who did not have the appropriate experience and remove them from the sample.

The second section contained the items for defined constructs, which consists of 47 questions regarding the respondents’ experience. This study consists of three groups of factors with a total of thirteen factors and thirty-nine items in addition to three outcome variables with three items for each outcome. Liker-type surveys are most recommended when relationships between constructs are complex and prevalent at the same time [29]. Multiple survey items were developed for each factor requiring the respondent to rate each item on a 5-point Likert scale of agreement, ranging from strongly disagree to strongly agree. The items were randomly shuffled to avoid respondents from determining the theoretical constructs. The full questionnaire is provided in Appendix A.

### 2.3. Sampling Approach

The potential participants for the survey were academic researchers or industry professionals who have participated in or observed the implementation of QMS in healthcare organizations. Due to the unavailable access to the database of all healthcare organizations’ personnel for the sample selection, convenience and purposive non-probability sampling are adopted since this study requires certain qualified members [30].

Exploratory Factor Analysis (EFA) is generally regarded as a technique for large sample sizes (N), with N = 50 as a reasonable absolute minimum [31]. Ref [32] characterized sample sizes above a sample size of at least 50 and not more than 100 subjects, which is adequate to represent and evaluate the psychometric properties of measures of social constructs [32]. Watkins et al. illustrated that when commonalities are high (greater than 0.60), and several items define each factor, sample sizes can actually be relatively small [33]. This study focused on the number of cases per variable (N:P), and recommendations varied from 3:1–6:1 [34] to 20:1 [35]. There is no official statistic of the potential respondents who have experience within the implementation of quality healthcare and can fit the purpose of this study. Consequently, since this study has an undefined target population, it aimed to achieve an N:P ratio of 5:1, which indicates that there should be at least five responses for every item in the model.

### 2.4. Pilot Test

A pilot study was conducted to test the survey with 18 subject participants who are experts in the area. The reliability of the variables was tested using Cronbach’s Alpha. The reliability values for the factors had different values with some factors scoring less than 0.5. Some factors can improve reliability when removing some items. Since the CA results alone are not decisive in redesigning the items, both CA results and pilot testers’ feedback were used to refine the items and improve the flow of the survey. The pilot study mainly helped in refining the statements and obtaining feedback from the testers about their experience in taking the survey, thus improving the clarity of the survey.

## 3. Results

The data collection resulted in 71 responses. The low response might be due to the highly specific scope of the research, where a unique system, such as QMS, is being studied in the setting of healthcare. The literature emphasizes that low response rates can be accepted given that the study takes steps to ensure the adequacy of the response [34]. Steps include ensuring that the survey instrument strictly applied the exclusion criteria to ensure that all survey respondents were appropriate. In addition, demographic analysis was performed to determine how participants’ different conditions can affect a QMS implementation. Full analysis can be provided upon request.

### 3.1. The Exploratory Factor Analysis (EFA)

EFA and Cronbach’s Alpha are used to refine the final set of factors. EFA is a clustering technique aiming to identify the underlying structure of factors, namely, their adequate grouping [35]. EFA was used to examine the proposed constructs’ validity and construct new factors from the items when needed. Multiple models were used to make the EFA process more effective and ensure adequate statistical power. Items were placed in models based on the operational research model grouping (Table 1). Separate EFA models were used for each of the major categories of factors. Items that hypothetically fall under the same main category were placed in the same model. For example, all management commitment, management training, and strategic planning items were placed in the same model that consisted of all items focused on management and planning. The five models included management and planning, culture, implementation resources, structure, and an outcome model. After performing the EFA as described, ten emergent factors yielded with their respective items as outlined in Table 3. For the EFA results, all factors have at least three items according to Thurstone’s recommendation for exploratory analysis [36]. The major EFA fit and the adequacy indices along with their acceptable values were reviewed in Table 4.

EFA is a highly interpretive approach, but multiple threshold metrics were used to guide the selection of items for each factor. The Pattern Matrix’s factor loadings should be close to or above 0.5 with 0 s-loadings below 0.3 [37,38]. Each item’s commonality should be above 0.4. Finally, the conceptual links among the items, supported by the co-occurrence network [27] and the reliability analysis results determined the final items for each emergent factor. All the models met all the acceptable values for the various indices as shown in Table 4.

The items in model 1 (Table 4) belonged to three major groups: Management training, commitment, and planning. This model’s EFA analysis identified the items in the same three factors: *Management Commitment, Management Training, and Strategic Planning.* The new factors’ items mostly matched the preliminary models except for MT1. MT1 was loaded into Factor 1 *Management Commitment* when it was originally with Factor 2 (Management Training). This repositioning of the MT1 item may be due to the focus on the management expectations of professionals regarding quality improvement. The item can be perceived as the role of the management rather than its competence. On another note, MT4, which described performance data used by management, fit into both Factors 1 and 2, due to the presence of performance and management in the item. Reliability measurements were obtained for the item in both factors to find the best fit. It was found that the reliability was enhanced with MT4 in Factor 3, thus it was added to Factor 3.

Model 2 (Table 4) contained nine items related to employees’ involvement, resistance to change, and communication. The EFA analysis identified two emergent factors for this model, Factor 4 (*QMSs’ Implementation Culture*) and Factor 5 (*Employee Focus*). Four items were loaded into Factor 4 that were initially related to employee involvement and resistance to change. The items represent culture and the human role in implementation, where the “resistance to change” item is related to culture. These two concepts were also associated according to the co-occurrence of factors. The result drew attention to this factor revolving around the *Culture* of *QMSs’ Implementation*. Three items were identified in Factor 5, and these items came from employee involvement and communication. Both the involvement of employees’ items and communication have focused on the personnel’s role in implementation. Moreover, the items can be attributed to enhancing the personnel’s ability to communicate and receive feedback and were found to be associated to the co-occurrence network, thus the factor was named *Employee Focus*. The EFA for model 2 dropped two items related to resistance to change and communication due to their low communality. This drop suggests that these items may not be factors themselves, but part of broader factors. The result confirms the new factors’ structure.

In model 3 (Table 4), ten items belonging to three factors were analyzed by EFA. The analysis loaded the items on the same three factors. All the items loaded into each factor matched the factors’ preliminary structure, showing a great extent of stability for these factors’ definitions. Therefore, the names of the factors remained the same as in the preliminary model. The stability confirms the preliminary build of the models and complies with the literature synthesis and expert study. Together, they contribute to the survey analysis’s total validity, particularly the face validity, which indicates that analogous items are loaded together on the same factor. Therefore, the names of the factors remained the same as in the preliminary model.

In model 4 (Table 4), eleven items were considered for the EFA. The items came from four different factors: Process and procedures, performance, audit and review, and customer focus. The EFA analysis of the eleven items loaded the items into two factors. Seven items of performance, customer focus, as well as audit and review factors loaded the items into one factor. The general theme of the seven items suggests the strong impact of customer focus on improvement. Moreover, the co-occurrence network [27] shows an adequate association between performance and customer focus. The results suggest the name “*Performance Improvement*” for the factor. The remaining four items were loaded into Factor 10. The items are composed of processes and procedure items, audits and reviews, and customer focus. The combination indicates a high resemblance to organizational structure, where audit and review items refer to protocol revision. Therefore, this group of items was found to show a strong resemblance to *Structure*. Finally, all the outcomes are loaded into one new factor (Table 4).

### 3.2. Reliability Analysis (Cronbach’s Alpha)

The resulting Cronbach’s Alpha value for all the factors and outcomes exceeded the lower threshold of 0.7, as shown in Table 5.

All the factors recorded high scores, including the outcome factor with a score of 0.908, indicating adequate reliability.

### 3.3. Analysis of Relationships

Analyzing the relationships among factors reveals the most significant factors connected to implementation outcomes. Correlation analysis and the factors’ effects on the outcomes using regression modeling were used to describe the relationships between factors. 

### 3.4. Regression Modeling

Linear multiple regression was used to assess the resulting set of emergent variables that yielded from the EFA. A multiple regression model is used to find the link between the emergent factors and the QMS implementation outcome. Multiple assumptions were examined to ensure the fitness and validity of the models [39].

**Figure 1:**
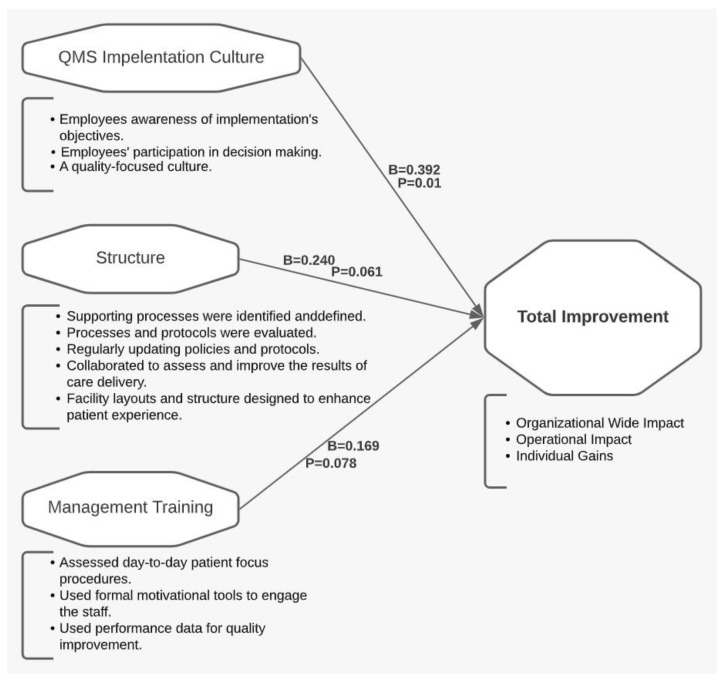
Implementation model.

To begin with, the model included the ten emergent factors as predictors and one outcome. The results are summarized in Table 6. The table shows that the model fit indices are relatively well met. The model had a significant F-test statistic using a 90% confidence interval, which indicates the probability of regression coefficient as zero. The significant results that are close to zero indicate a very low probability with a zero-regression coefficient, thus providing evidence for the fitness of the model. The critical factors are Factor 2 (Management Training), Factor 4 (QMSs’ Implementation Culture), and Factor 10 (Structure), as represented in the final model shown in Figure 1. Detailed results of the regression models can be provided upon request.

### 3.5. Investigation of Interrelationships among Factors

The Causal Loop Diagram (CLD) is an approach used to show the feedback structure and can describe the causal effects between the identified factors [40,41]. This study develops a CLD using a series of multiple linear regression models of the factors that affect QMS implementation success. Hypothesized relationships among success factors were analyzed to find the factors’ connections. The regression analysis considered a series of multiple regression analysis where factors were modeled against one another. As an example, one model had Management Training as the dependent variable and the other factors were the predictors. In total, ten regression models were created (i.e., one model for each of the emergent factors). All required assumptions and model fitness were checked.

**Figure 2:**
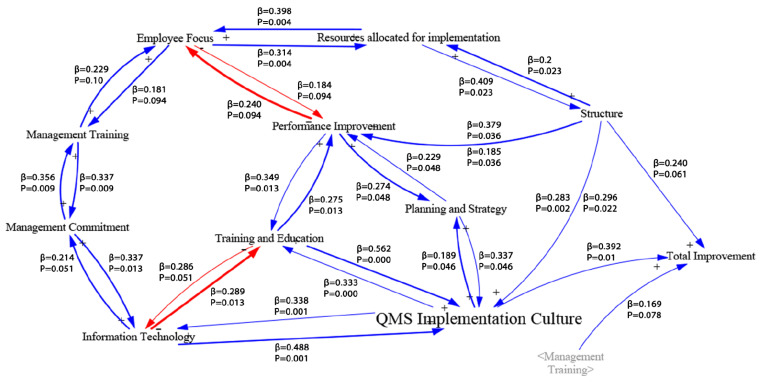
CLD model.

Next, each of the ten regression models was developed using SPSS software and the remaining assumptions and measures of model fit were evaluated. Then, the results were used to develop the resulting hypothesized CLD. The resulting regression models were used to develop the CLD model, as shown in Figure 2 below.

The hypothesized CLD includes arrows showing the direction of the relationships and the type of effect. It can be observed that there is self-reinforcing feedback in all loops except for the relationship of *Information Technology* on *Training and Education, Employee Focus* on *Performance Improvement.* Although the results are not expected, they can be justified by considering the effects over time. The results show many significant relationships between the variables. This could be considered unusual compared to studies about critical success factors, but most of the results were expected [40,42]. Shadowing of the CSFs was used to fully view all the CSFs connections to the outcome, as indicated by the italicized labels with a grey font. 

## 4. Discussion

The results of the EFA models were not surprising, with many factors retaining their original structure. This confirms the preliminary design of the model and aligns with the findings of the literature synthesis and the expert study, contributing to the survey analysis’s total validity and the EFA analysis’s validity, particularly the face validity, which indicates that similar nature items are loading together on the same factor. All nine items in the outcome model are loaded into one outcome, as shown in Table 4. This can be attributed to the difficulty in detecting the impact of QMSs’ implementation that respondents perceived similarly.

The regression results suggest that implementing a culture where quality is centered within the organization has a significant effect on the successful QMSs’ implementation conforming with what has been referred to by the literature [43]. For QMSs to succeed, a collaborative and corporate organizational culture should be supported by long-term management, employee commitment, organizational learning, and training. Management training is essential as it is the main facilitator for implementation [44]. Moreover, the results showed that a solid organizational structure is needed to support the successful implementation of a QMS.

The model represents an answer to the major research questions about the CSFs responsible for a successful implementation of QMS in connection to the implementation’s main outcome. The structured and systematic technique used, beginning with refining the factors followed by the multiple regression modeling, ensured the final model’s validity and accuracy. Moreover, the CSFs are in conformance with the factors for general change initiative in healthcare. Kasha et al., 2014 found that improving quality embedment in the healthcare organization environment is one of the most critical success factors for change. They stated that this is one of the unique success factors for healthcare that is not regularly found in change models [45]. These factors’ uniqueness can be proven by comparing them to literature in other industries, where studies have found the quality culture to be adequately instilled within the organizations [46]. In addition, the model confirms many findings of implementation of different systems in healthcare, such as information systems, where the main consideration for implementation was to train staff [47]. Other industries have also emphasized the importance of training managers and leaders on quality principles [48]. In the literature, critical success factors of QMS implementation did not report the structure as a CSF [6,49,50,51]. Finding the structure as one of the CSFs is aligned with the initial findings of recent reports about the silo mentality, which is a source of conflict in healthcare structure [52,53]. The result of this study can suggest that having more than one quality entity in the organization can challenge the total improvement. The CSFs that resulted from the regression were mainly aligned with the correlation analysis. Both the structure and the QMS implementation factors were the top two correlated factors with the outcome, but the management training was not highly correlated with the outcome.

Furthermore, the CLD has presented other central factors to the implementation process, although they were not deemed critical for the outcomes. For example, *Performance Improvement* is critically connected to four other factors with a solid connection to the CSF *Structure.* Another strong connection was with the *Training and Education* factor, which is consistent with previous literature assumptions that indicated the need for proper quality improvement skills to perform any improvement initiatives [54]. This can be achieved using systemized and well-targeted training and education programs. This notion sheds light on the *Training and Education* factor, which was also connected to three other factors, including a strong relationship to *QMS Implementation Culture*. The connection can be verified by noting one of the *QMS Implementation Culture* components, resistance to change, where education about QMSs’ role and encouraging its principles can make employees inherently eager to adopt the QMS principles. One final example of a central factor is the *Information Technology* factor. Since this factor is responsible for providing data and measuring performance, it was expected to have a direct connection to *Performance Improvement*; however, more critical connections were found for *Management Commitment*. This result can be due to how the CLD model is developed, which is based on multiple relationships between the factors. Therefore, this creates a chain of effect, where one factor affects the other and this factor affects another factor. The CLD model provided essential information about the interactions among factors as well as another dimension of significance. The model was able to show which factors are central to a group of factors providing additional insights beyond the CSFs for positive outcomes.

The investigations of implementation success factors in the literature were primarily qualitative or used the simple descriptive analysis. Few studies have used multiple advance statistical analyses and identified factors related to organizational structure, including procedures, working guidelines, and resources, which were found to be important for the total improvement outcome in this research [28,44,55]. Aburayya, Alshurideh [25] has performed advanced statistical analysis, including factor analysis, but the research lacked the relationship among factors.

Interestingly, none of the quantitative studies in the literature found Management Training crucial for the implementation. The previous quantitative studies confirm the variation in the factors studied, their terminology, and the context in which the studies were conducted. The results of the model testing study matched the results provided by the literature. This is probably natural since the underlying concepts that form the survey are the most commonly identified factors in the literature.

## 5. Conclusions

Initially, the study developed an operational research model with thirteen preliminary factors on the basis of a literature review and expert study. EFA analysis and multiple linear regression helped refine the factors and analyze their effect on implementation. Multiple emergent factors matched the initial factors. Factors, such as *Strategic Planning*, *Training and Education, Resources Allocated*, and *Information Technology,* had the same items from the preliminary model. While factors, such as *Management Commitment* and *Management Training,* had only a slight difference (i.e., only one item changed). The primary factors of *Employee Involvement, Customer Focus, Resistance to Change, Audit, Communication, Performance, and Processes and Procedures* were highly affected. They yielded a new group of factors that were named: *QMSs’ Implementation Culture, Employee Focus, Performance Improvement,* and *Structure.* The regression model found three critical success factors that are linked directly to the outcome of success. The factors were *Implementation Culture*, *Management Training,* and *Structure*. The CSFs agreed with general change and systems implementation in healthcare, where improving system embeddedness in the healthcare organization environment was one of the most critical success factors for change. Comparing this list of CSFs to other sectors proves how the study resulted in more healthcare-related CSFs. The three variables have covered a wide spectrum of items in the survey and have a solid base in the literature, supporting the survey instrument’s validity and providing significant insights into the factors responsible for implementation. Moreover, the survey instrument was able to find the correlations among factors and perform regression modeling that helped initiate the CLD of the factors’ relationships. The results show significance in all the relationships between the variables. This could be considered unusual compared to studies about critical success factors, but most of the results were expected [40,42]. Shadowing of the CSFs was used to fully view all the critical success factors connections to the outcome.

The survey analysis has provided quantitative evidence about the factors and the outcomes of implementation success, which will contribute to the literature in this area that sorely lacks the depth of recent empirical evidence. This research presented empirically operationalized models of understanding for both QMS and implementation success. This process provides a solid, clear basis for any build-up in future research and allows for an enhanced background for perceiving general studies’ results. Finally, the survey study was conducted with a broad sample of healthcare quality experts from various roles with experience in applying different types of QMS approaches and in multiple healthcare settings. This quality in the sampling enhanced this research’s generalizability. The multi-item construct survey that tested the model provided a robust construct refinement and allowed further examination through advanced statistical techniques.

The implication from the research comes from the most significant factor that the study identified: The *QMS Implementation Culture.* In particular, the need to understand that the working environment with all stakeholders’ behaviors and attitudes toward the implementation poses a crucial effect on success. Therefore, acknowledging quality as a routine rooted in all aspects of the process will alleviate the difficulties in implementing the QMS. Moreover, quality thinking can ease the implementation of improved processes and procedures and reshaping them to be patient focused. The principal key practical implication is that the implementation of QMS is an installment of a system and a change of mindset. Furthermore, the comprehensive results of this research can assist in a deeper understanding and a high level of planning.

The limitations of this research are related to the construction of the survey and the research sample. The survey was developed based on a rigorous review of the literature and an expert study. However, the data related to measuring the potential success factors (independent variables) and outcome variables (dependent variables) were collected from the same source, which may introduce a common method bias [56]. Another main limitation was related to the size of the sample. Different circumstances may have affected the data collection and hindered our ability to reach out to participants in the healthcare sector. Although the small sample might affect the strength and validity of the analysis, the study strived to mitigate this risk using techniques that are suitable for data analysis of smaller samples. Performing EFA separately for each model of factors was a technique that helped address this risk by achieving an adequate N:P ratio.

Additionally, the measures that emerged from this research, the ten success factors, should include further analysis to ensure their validity and reliability across a variety of situations and contexts. All participants stated experiencing a successful implementation, which might be due to the survivorship bias. In survivorship bias, people tend to report only the successful cases, while leaving the unsuccessful cases unevaluated, which results in incomplete conclusions. This form of bias could produce a lack of full perspective about the QMS implementation in the case of failure. The study results are based primarily on US insights that may not be applicable in other social contexts. However, it provides results that can be highly related to a certain context.

## Figures and Tables

**Table 1:** Preliminary factors and their frequency.

Factor Group	Factor	Acronyms and Item No.	Sub-Code	Integrated Frequency
Management	Management Commitment	MC1	Management Involvement	9%
		MC2	Management Oversight	
		MC3	Engagement of Top Leaders	
	Management Training	MT1	Clear Expectation	4%
		MT2	Compliance Assessment	
		MT3	Motivate Staff	
		MT4	Analyze Data	
Organization Culture	Employees Involvement	EI1	Employee Engagement	10%
		EI2	Reward	
		EI3	Awareness of QMS	
		EI4	Employee Satisfaction	
		EI5	Feedback Role	
	Resistance to Change	RC1	Adoption	6.5%
		RC2	Quality-Focused	
	Training and Education	TE1	Quality Education	11%
		TE2	Learning Evaluation	
		TE3	Competency	
	Communication	C1	Deliver Expectation	10.5%
		C2	Communication Among Levels	
	Resources Allocated for Implementation	R1	Support Process	14.5%
		R2	Funding	
		R3	Adequate Staffing	
		R4	Dedicate Time	
	Information Technology	IT1	Information Management System	5.5%
		IT2	Data for Improvement	
		IT3	Data Analysis	
Structure	Processes and Procedures	PP1	Identify Process	6.5%
		PP2	Evaluate Process	
		PP3	Update Protocols	
	Performance	PER1	Complaints’ Evaluation	3.5%
		PER2	Performance Indicators	
		PER3	Continuous Improvement	
	Customer Focus	CF1	Patient Focus	5%
		CF2	Patient Feedback	
		CF3	Patient Experience	
	QMSs’ Review and Audit	AUD1	Internal Audit	4.5%
		AUD2	Collaboration	
	Strategic Planning	SP1	Long-term Goals	16.5%
		SP2	Align Strategies	
		SP3	Quality Integration	

**Table 2:** Preliminary outcomes and their frequency.

Outcome	Acronym	Sub-Code	Aggregate Frequency
Organizational Wide Impact	OI1	ORG Performance	35%
	OI2	ORG Achievement	
	OI3	Responsibility Sense	
Operational Impact	OP1	Service Improvement	30%
	OP2	Processes Redesign	
	OP3	Enhanced Communication	
Individual gains	IG1	Raised Commitment	35%
	IG2	Improved Motivation	
	IG3	Increased Satisfaction	

**Table 3:** Emergent factors and their items.

Model	Emergent Factor	Items						
**Model 1**	Factor 1 (Management Commitment)	MC1	MC2	MC3	MT1			
	Factor 2 (Management Training)	MT2	MT3	MT4				
	Factor 3 (Planning and Strategy)	SP1	SP2	SP3				
**Model 2**	Factor 4 (QMSs’ Implementation Culture)	EI5	EI3	RC2				
	Factor 5 (Employee Focus)	C2	EI2	EI4				
**Model 3**	Factor 6 (Resources allocated for implementation)	R1	R2	R3	R4			
	Factor 7 (Training and Education)	TE1	TE2	TE3				
	Factor 8 (Information Technology)	IT1	IT2	IT3				
**Model 4**	Factor 9 (Performance Improvement)	PP3	PER3	CF1	AUD1	PER2	CF2	PER1
	Factor 10 (Structure)	PP2	PP3	PP1	AUD2	CF3		
**Outcome**	Outcome (Total Improvement)	OI1	OI2	OI3	OP1	OP2	OP3	IG1
		IG2	IG3					

**Table 4:** EFA fit of emergent factors models.

Model	Initial Factor	K	N:P	KMO	Cumulative Variance	Determinant	Number of New Factors
Model 1: Management	Management Commitment Management Training Strategic Planning	10	7:1	0.837	66%	0.002	3
Model 2: Culture	Employees Involvement Resistance to Change Communication	9	8:1	0.805	55%	0.023	2
Model 3: Implementation resources	Resources Allocated for Implementation Training and Education Information Technology	10	7:1	0.809	63%	0.004	3
Model 4: Structure	Processes and Procedures Performance Customer Focus Audit	11	7:1	0.833	52%	0.001	2
Model 5: Implementation outcomes	Organizational Wide Impact Organizational Performance Individual Gain	9	8:1	0.833	52%	0.001	1

**Table 5:** Emergent factors’ reliability.

Factor	Reliability
Management Commitment	0.903
Management Training	0.740
Planning and Strategy	0.856
QMSs’ Implementation Culture	0.841
Employee Focus	0.716
Resources Allocated for Implementation	0.852
Training and Education	0.814
Information Technology	0.818
Performance Improvement	0.875
Structure	0.749
Implementation Success Outcomes	0.908

**Table 6:** Multiple linear regression model.

Model		STD. Coefficients (BETA)	B	T	SIG
R^2^	0.713	Constant		1.134	0.0261
ADJ.R^2^	0.666	Factor 1 (Management Commitment)	0.063	0.645	0.522
STD Error	0.418	**Factor 2 (Management Training)**	0.169	1.795	0.078
Durbin−Watson	2.3	Factor 3 (Planning and Strategy)	−0.043	−0.389	0.699
**ANOVA**		**Factor 4 (QMSs’ Implementation Culture)**	0.392	2.643	0.010
F	14.936	Factor 5 (Employee Focus	−0.020	−0.187	0.852
Sig	0.000	Factor 6 (Resources Allocated For Implementation)	0.161	1.593	0.116
		Factor 7 (Training and Education)	0.185	1.645	0.105
		Factor 8 (Information Technology)	−0.149	−1.242	0.219
		Factor 9 (Performance Imrovment)	0.146	1.444	0.154
		**Factor 10 (Structure)**	0.240	1.909	0.061

## Data Availability

The data presented in this study are available on request from the corresponding author. The data are not publicly available due to their use in further analysis.

## Appendix A. Survey Protocol

***“Factors that Affect the Successful Implementation of Quality Management Systems in Healthcare”***

You are being invited to take part in a research study. Whether you take part is up to you. The purpose of this study is to investigate the factors that influence the Quality Management System (QMS) implementation in healthcare organizations as part of a doctoral study focused on improving QMS implementation success. Identifying these factors and evaluating their relative impact on implementation success will support the research team in their efforts to develop strategies to improve QMS implementation in practice. You have been identified as a potential participant in this *survey*, which takes approximately 20–25 min to complete. It is important to note that the study results will be strictly confidential and only aggregate results will be used for the analysis and dissemination, ensuring that no individual participants are identifiable.

**Study contact, for questions about the study or to report a problem:** If you have questions, concerns or complaints please contact:

Mustafa Rawshdeh, Graduate Student, Industrial Engineering and Management Systems Program, College of Engineering and Computer Science at (407) 864-3534

or Dr. Heather Keathley, Faculty Supervisor, Department of Industrial Engineering and Management Systems at (407) 823-4745 or by email at heather.keathley@ucf.edu.

**IRB contact, about your rights in the study or to report a complaint:** Research at the University of Central Florida involving human participants is carried out under the oversight of the Institutional Review Board (UCF IRB). This research has been determined to be exempt from IRB review unless changes are made. For information about the rights of people who take part in research, please contact the Institutional Review Board, University of Central Florida, Office of Research and Commercialization, 12201 Research Parkway, Suite 501, Orlando, FL 32826-3246 or by telephone at (407) 823-2901.

**Principal Investigator:** Mustafa Rawshdeh

**Faculty Supervisor:** Heather Keathley, Ph.D.

Would you like to participate in this survey? Please note that you may exit now and return later if you would like. Contact the researchers regarding any questions, comments or concerns.

Yes, I would like to complete the survey.

No, I do not want to participate in this survey.

***Skip To: Beginning of Survey If Factors that Affect the Successful Implementation of Quality Management Systems in Healthcare Y = Yes, I would like to complete the survey***
***Skip To: End of Survey If Factors that Affect the Successful Implementation of Quality Management Systems in Healthcare N = No, I do not want to participate in this survey***

Thank you for agreeing to participate in this study. This survey consists of two sections:

- General demographic information.
- Likert-style questions to assess factors and outcomes of implementation based on your experience.

Healthcare organizations, similar to many other industries, often face significant challenges during QMS implementation. Identifying the factors that affect successful QMS implementation will support healthcare professionals in developing strategies to improve the implementation process, allowing organizations to obtain the potential benefits of these systems.

Below are some terms that are relevant to this study:

**A Quality Management System (QMS).** A management system used to monitor and improve all components of an organization from a quality perspective. Unlike medical quality control procedures, such as infection control, QMS focuses on process quality and improving organizational performance and effectiveness. Common frameworks include ISO 9000 and 9001, Total Quality Management (TQM), and the Baldrige Criteria.

**Implementation.** Installing a system into action to achieve the required standards and fulfill the awaited goals. This process includes the initial execution of the completed design as well as deployment throughout the organization.

**Factors**. All barriers, obstacles, enablers or any issues that can affect the implementation.

Q1.1 Instructions: This section consists of a few questions to gain more information about your background and provides the context for your responses. Consider the last QMS implementation that you participated in or observed in healthcare.
Q1.2 What is your Current Position?
Quality Professional
Administrative
Medical Staff (Physician, Nurses, etc.)
Researcher
Other ________________________________________________
Q1.3 How many years of experience do you have in quality management?
Less than 2 years
3–5 years
6–10 years
More than 10 years
Q1.4 When was the last time that you participated in or observed the implementation of a new or significantly redesigned Quality Management System (QMS) in a healthcare organization?
1–2 years
3–5 years
More than 5 years ago
I have never observed or implemented a QMS in a healthcare organization.
***Skip To: End of Survey If When was the last time that you participated in or observed the implementation of a new or modified QMS = I have never observed or implemented a QMS in a healthcare organization***
Q1.5 Which type of healthcare did you experience or observe QMS implementation in?
Public
Private
Q1.6 Which area(s) of healthcare was the focus on the QMS implementation?
Hospital
College Medical Center
Single department (i.e., operating room)
Outpatient care center (i.e., urgent care)
Physician’s offices
Medical and Diagnostic laboratories
Other ________________________________________________
Q1.7 Which best describes the size of the healthcare organizations?
Small: Fewer than 99 employees
Medium: 100 to 499 employees
Large: 500 to 2499 employees
Corporate: More than 2500 employees
Q1.8 Which of the following accreditation/certifications/philosophies were used to develop the QMS that you helped to implement? (select all that apply)
ISO 9001 (International Standards Organization)
EFQM (European Foundation for Quality Management)
MBNQA (Malcolm Baldrige National Quality Award)
TQM (Total Quality Management)
None/customized system
Other ________________________________________________
Q1.9 What role (or roles) did you serve during the QMS implementation? (select all that apply)
Team Leader
Facilitator
Champion
Process Owner
Team Member
Management
Observer/Studying
Other ________________________________________________
Q1.10 In general, how successful was the last implementation that you participated in or observed?
Extremely successful
Very successful
Moderately successful
Slightly successful
Not successful at all
Q2.1 Instructions: Below are questions regarding QMS implementation factors that are studied in the literature. It is important to note that we are interested in your experiences or opinions.

**Q2.2 To what Extent do You Agree or Disagree with the Following Statements?**	**Strongly Disagree**	**Disagree**	**Neither Agree nor Disagree**	**Agree**	**Strongly Agree**
Management was involved in quality improvement activities.	1	2	3	4	5
Management was committed to high-quality services.	1	2	3	4	5
Management monitored the execution of quality improvement plans.	1	2	3	4	5
Management clearly communicated expectations for care professionals regarding quality improvement.	1	2	3	4	5
Management used performance data for quality improvement.	1	2	3	4	5
Management assessed care-professionals’ compliance with day-to-day patient safety procedures.	1	2	3	4	5
Management used formal motivational tools to engage the staff.	1	2	3	4	5

Q3.1 Instructions: Below are questions regarding QMS implementation factors that are studied in the literature. It is important to note that we are interested in your experiences.

**Q3.2 To what Extent do You Agree or Disagree with the Following Statements?**	**Strongly Disagree**	**Disagree**	**Neither Agree nor Disagree**	**Agree**	**Strongly AGREE**
Differences in patients’ expectations and actual service was communicated to employees.	1	2	3	4	5
Employees’ feedback was part of decision making.	1	2	3	4	5
Employees were involved in quality activities.	1	2	3	4	5
Employees were aware of the QMS implementation objectives.	1	2	3	4	5
Adequate quality education and training were provided when needed.	1	2	3	4	5
Learning and comprehension of quality tools and principles were evaluated.	1	2	3	4	5
Employees assigned to QMS tasks were competent.	1	2	3	4	5
Employees’ satisfaction with the QMS was measured.	1	2	3	4	5
Employees were appropriately recognized or rewarded for engagement in the implementation effort.	1	2	3	4	5
There were sufficient resources to support quality projects/processes.	1	2	3	4	5
There were adequate staff in support of the QMS.	1	2	3	4	5
There was adequate funding for QMS purposes.	1	2	3	4	5
Data generated from information management systems were used for improvement.	1	2	3	4	5
The organization used an information management system.	1	2	3	4	5
Quality data and information were analyzed.	1	2	3	4	5
Communication between different levels of management was effective.	1	2	3	4	5
Adequate time was allocated for staff to conduct quality tasks.	1	2	3	4	5
The organization had a quality-focused culture.	1	2	3	4	5
Staff easily adopted quality concepts.	1	2	3	4	5

Q4.1 Instructions: Below are questions regarding QMS implementation factors that are studied in the literature. It is important to note that we are interested in your experiences.

**Q4.2 To what Extent do You Agree or Disagree with the Following Statements?**	**Strongly Disagree**	**Disagree**	**Neither Agree nor Disagree**	**Agree**	**Strongly Agree**
Supporting processes were identified and defined.	1	2	3	4	5
The organization regularly updated their policies and protocols.	1	2	3	4	5
Processes and protocols were regularly evaluated.	1	2	3	4	5
The organization had a formal process to continuously revise the QMS.	1	2	3	4	5
The organization considered customer needs in process improvement activities.	1	2	3	4	5
The organization regularly evaluated the QMS function (i.e., internal audits).	1	2	3	4	5
Different roles collaborated to assess and improve the results of care delivery.	1	2	3	4	5
Performance indicators were compared with other healthcare organizations to identify opportunities for improvement.	1	2	3	4	5
Patients were periodically requested to give their opinions on the care provided.	1	2	3	4	5
A periodical evaluation of complaints was used to implement improvements.	1	2	3	4	5
The organization pursued long-term organizational goals and policies.	1	2	3	4	5
The organization integrated quality in the strategic plan.	1	2	3	4	5
Policies and strategies were developed according to current and future needs.	1	2	3	4	5
Facility layouts and structure were designed to enhance patient experience.	1	2	3	4	5

Q5.1 Instructions: Below are questions regarding the primary benefits of successfully implementing a QMS. It is important to note that we are interested in your experiences.

**Q5.2 To what Extent do You Agree or Disagree that QMS Implementation Resulted in:**	**Strongly Disagree**	**Disagree**	**Neither Agree nor Disagree**	**Agree**	**Strongly Agree**
Improved organizational performance.	1	2	3	4	5
Achievement of related accreditation or awards.	1	2	3	4	5
Increased quality of services provided.	1	2	3	4	5
Developing a sense of responsibility sharing.	1	2	3	4	5
Enhanced stakeholders’ satisfaction.	1	2	3	4	5
Redesigned procedures and standards.	1	2	3	4	5
Enhanced communication among different levels of employees.	1	2	3	4	5
Increased employee organizational commitment.	1	2	3	4	5
Increased employee motivation.	1	2	3	4	5

Thank you for your participation in this study. If you have any remaining questions or concerns, please contact the researcher: Mustafa Rawshdeh at Rawshdeh@knights.ucf.edu.

## References

[1] A’Aqoulah A., Kuyini A.B., Ajlouni M.T. (2016). Addressing Quality Management System Obstacles in Jordanian Hospitals. Int. Bus. Res..

[2] World Health Organization (2019). Improving Healthcare Quality in Europe Characteristics, Effectiveness and Implementation of Different Strategies: Characteristics, Effectiveness and Implementation of Different Strategies.

[3] Wagner C., De Bakker D.H., Groenewegen P. (1999). A measuring instrument for evaluation of quality systems. Int. J. Qual. Health Care.

[4] Psychogios A.G., Atanasovski J., Tsironis L.K. (2012). Lean Six Sigma in a service context: A multi-factor application approach in the telecommunications industry. Int. J. Qual. Reliab. Manag..

[5] Hietschold N., Reinhardt R., Gurtner S. (2014). Measuring critical success factors of TQM implementation successfully—A systematic literature review. Int. J. Prod. Res..

[6] Aquilani B., Silvestri C., Ruggieri A., Gatti C. (2017). A systematic literature review on total quality management critical success factors and the identification of new avenues of research. TQM J..

[7] Donabedian A. (1988). The quality of care: How can it be assessed?. JAMA.

[8] Tamer G., Çetinkaya H. (2018). Impact of quality management system on health institutions. Health Care Acad. J..

[9] Moldovan F., Blaga P., Moldovan L., Bataga T. (2022). An Innovative Framework for Sustainable Development in Healthcare: The Human Rights Assessment. Int. J. Environ. Res. Public Health.

[10] Abdallah A. (2014). Implementing quality initiatives in healthcare organizations: Drivers and challenges. Int. J. Health Care Qual. Assur..

[11] Committee H.T. (2016). A Hospital-Based Healthcare Quality Management System Model.

[12] Sousa R., Voss C. (2002). Quality management re-visited: A reflective review and agenda for future research. J. Oper. Manag..

[13] Moukhafi S. (2021). Hospital quality management: A historical foundation. Rev. Econ. Gest. Soc..

[14] Bortolotti T., Boscari S., Danese P., Suni H.A.M., Rich N., Romano P. (2018). The social benefits of kaizen initiatives in healthcare: An empirical study. Int. J. Oper. Prod. Manag..

[15] Parast M.M., Golmohammadi D. (2019). Quality management in healthcare organizations: Empirical evidence from the baldrige data. Int. J. Prod. Econ..

[16] Kelly D.L. (2007). Applying Quality Management in Healthcare: A Systems Approach.

[17] Alaraki M.S. (2014). The Impact of Critical Total Quality Management Practices on Hospital Performance in the Ministry of Health Hospitals in Saudi Arabia. Qual. Manag. Health Care.

[18] Unger K.L. (2013). An Investigation into the Effects of Winning the Malcolm Baldrige National Quality Award on the Performance of Hospitals/Healthcare Systems.

[19] Aburayya A., Alshurideh M., Marzouqi A.A., Diabat O.A., Alfarsi A., Suson R., Bash M., Salloum S.A. (2020). An empirical examination of the effect of TQM practices on hospital service quality: An assessment study in UAE hospitals. Syst. Rev. Pharm..

[20] Wardhani V., Utarini A., van Dijk J.P., Post D., Groothoff J.W. (2009). Determinants of quality management systems implementation in hospitals. Health Policy.

[21] Ali Mohammad M. (2014). Why TQM does not work in Iranian healthcare organisations. Int. J. Health Care Qual. Assur..

[22] Ali Mohammad M. (2013). Obstacles to TQM success in health care systems. Int. J. Health Care Qual. Assur..

[23] Hasanali F. (2002). Critical Success Factors of Knowledge Management. http://www.providersedge.com/docs/km_articles/Critical_Success_Factors_of_KM.pdf.

[24] Mosadeghrad A.M., Jaafaripooyan E., Dehnavi H. (2022). Critical success factors of hospitals: A qualitative study. J. Iran. Inst. Health Sci. Res..

[25] Aburayya A., Alshurideh M., Al Marzouqi A., Al Diabat O., Alfarsi A., Suson R., Salloum S.A., Alawadhi D., Alzarouni A. (2020). Critical Success Factors Affecting the Implementation of TQM in Public Hospitals: A Case Study in UAE Hospitals. Syst. Rev. Pharm..

[26] Claver E., Tarí J.J., Molina J.F. (2003). Critical factors and results of quality management: An empirical study. Total Qual. Manag. Bus. Excel..

[27] Rawshdeh M. (2021). Factors that Affect the Successful Implementation of Quality Management Systems in Healthcare.

[28] Mosadeghrad A.M. (2015). Developing and validating a total quality management model for healthcare organisations. TQM J..

[29] Joshi A., Kale S., Chandel S., Pal D.K. (2015). Likert scale: Explored and explained. Curr. J. Appl. Sci. Technol..

[30] Etikan I., Musa S.A., Alkassim R.S. (2016). Comparison of Convenience Sampling and Purposive Sampling. Am. J. Theor. Appl. Stat..

[31] de Winter J.C.F., Dodou D., Wieringa P.A. (2009). Exploratory Factor Analysis with Small Sample Sizes. Multivar. Behav. Res..

[32] Sapnas K.G., Zeller R.A. (2002). Minimizing Sample Size When Using Exploratory Factor Analysis for Measurement. J. Nurs. Meas..

[33] Watkins M.W. (2018). Exploratory Factor Analysis: A Guide to Best Practice. J. Black Psychol..

[34] Baruch Y., Holtom B.C. (2008). Survey response rate levels and trends in organizational research. Hum. Relat..

[35] Hair J.F. (1998). Multivariate Data Analysis.

[36] Andrich D. (1978). Relationships Between the Thurstone and Rasch Approaches to Item Scaling. Appl. Psychol. Meas..

[37] Thompson B. (2017). Exploratory and Confirmatory Factor Analysis: Understanding Concepts and Applications. Appl. Psychol. Meas..

[38] Cohen P., West S.G., Aiken L.S. (2013). Applied Multiple Regression/Correlation Analysis for the Behavioral Sciences.

[39] Oladimeji O. (2019). Developing Causal Relationships for the Performance Measurement Implementation Process. Proceedings of the International Annual Conference of the American Society for Engineering Management.

[40] Sterman J.D. (2001). System Dynamics Modeling: Tools for Learning in a Complex World. Calif. Manag. Rev..

[41] Littlejohns L.B., Baum F., Lawless A., Freeman T. (2018). The value of a causal loop diagram in exploring the complex interplay of factors that influence health promotion in a multisectoral health system in Australia. Health Res. Policy Syst..

[42] Rad A.M.M. (2006). The impact of organizational culture on the successful implementation of total quality management. TQM Mag..

[43] Buciuniene I., Malciankina S., Lydeka Z., Kazlauskaite R. (2006). Managerial attitude to the implementation of quality management systems in Lithuanian support treatment and nursing hospitals. BMC Health Serv. Res..

[44] Kash B.A., Spaulding A., Johnson C.E., Gamm L. (2014). Success factors for strategic change initiatives: A qualitative study of healthcare administrators’ perspectives. J. Health Manag..

[45] Bravi L., Murmura F., Santos G. (2019). The ISO 9001: 2015 quality management system standard: Companies’ drivers, benefits and barriers to its implementation. Qual. Innov. Prosper..

[46] Creswell J.W., Klassen A.C., Clark V.L.P., Smith K.C. (2011). Best Practices for Mixed Methods Research in Health Sciences.

[47] Seetharaman A., Sreenivasan J., Boon L.P. (2006). Critical Success Factors of Total Quality Management. Qual. Quant..

[48] Sila I., Ebrahimpour M. (2003). Examination and comparison of the critical factors of total quality management (TQM) across countries. Int. J. Prod. Res..

[49] Sila I., Ebrahimpour M. (2002). An investigation of the total quality management survey based research published between 1989 and 2000. Int. J. Qual. Reliab. Manag..

[50] Karuppusami G., Gandhinathan R. (2006). Pareto analysis of critical success factors of total quality management: A literature review and analysis. TQM Mag..

[51] Alves J., Meneses R. Silos mentality in healthcare services. Proceedings of the 11th Annual Conference of the EuroMed Academy of Business.

[52] Caseiro J., Meneses R. Is Silo Mentality Relevant in Healthcare? The Healthcare Professional’s View. Proceedings of the STRATEGICA.

[53] Mosadeghrad A.M. (2014). Essentials of total quality management: A meta-analysis. Int. J. Health Care Qual. Assur..

[54] Adeoti J.O. (2011). Total quality management (TQM) factors: An empirical study of Kwara state government hospitals. Stud. Ethno-Med..

[55] Friedrich T.L., Byrne C.L., Mumford M.D. (2009). Methodological and theoretical Considerations in survey research. Leadership Q..

[56] Cattell R. (2012). The Scientific Use of Factor Analysis In Behavioral and Life Sciences.

